# Neural progenitor cell-derived nanovesicles promote hair follicle growth via miR-100

**DOI:** 10.1186/s12951-020-00757-5

**Published:** 2021-01-11

**Authors:** Lei Cao, Tian Tian, Yuanbo Huang, Shiqin Tao, Xiaohong Zhu, Mifang Yang, Jing Gu, Guangdong Feng, Yinni Ma, Rushan Xia, Wenrong Xu, Lei Wang

**Affiliations:** 1 Department of Dermatology, The Affiliated Wuxi No. 2 People’s Hospital of Nanjing Medical University, Wuxi, 214000 Jiangsu China; 2 Department of Neurobiology, Nanjing Medical University, Nanjing, 211166 Jiangsu China; 3 Department of Dermatology, The Affiliated Wuxi People’s Hospital of Nanjing Medical University, Wuxi, 214000, Jiangsu China; 4 Jiangsu Key Laboratory of Oral Diseases, Nanjing Medical University, Nanjing, 210029 Jiangsu China; 5 Institute of Stomatology, The Affiliated Hospital of Stomatology, Nanjing Medical University, Nanjing, 210029 China

**Keywords:** Hair growth, Nanovesicles, Extracellular vesicles

## Abstract

**Background:**

Accumulating evidence shows that mesenchymal stem cell-derived extracellular vesicles (EVs) hold great promise to promote hair growth. However, large-scale production of EVs is still a challenge. Recently, exosome-mimetic nanovesicles (NV) prepared by extruding cells have emerged as an alternative strategy for clinical-scale production. Here, ReNcell VM (ReN) cells, a neural progenitor cell line was serially extruded to produce NV.

**Results:**

ReN-NV were found to promote dermal papilla cell (DPC) proliferation. In addition, in a mouse model of depilation-induced hair regeneration, ReN-NV were injected subcutaneously, resulting in an acceleration of hair follicle (HF) cycling transition at the site. The underlying mechanism was indicated to be the activation of Wnt/β-catenin signaling pathway. Furthermore, miR-100 was revealed to be abundant in ReN-NV and significantly up-regulated in DPCs receiving ReN-NV treatment. miR-100 inhibition verified its important role in ReN-NV-induced β-catenin signaling activation.

**Conclusion:**

These results provide an alternative agent to EVs and suggest a strategy for hair growth therapy.

## Background

Hair loss, characterized by shorter anagen and longer telogen phases of hair follicles (HF), is a common medical problem which may cause both cosmetic and psychological problems. Minoxidil, finasteride, platelet-rich plasma (PRP), low-level laser therapy (LLLT), stem cell therapy, and HF transplantation are current treatment approaches. Unfortunately, all of these treatments have limitations. According to previous studies, minoxidil may cause high rate of adverse effects, such as burning or pruritus at the application site, allergic contact dermatitis [1] and cardiovascular effects [2]. Finasteride was associated with rare complications of decreased libido [3], gynecomastia [4] and psychologic impairments [5]. LLLT may bring about adverse events including dry skin, pruritus, and scalp tenderness [6]. PRP has the limitation of no consensus regarding exact concentration, dosing parameters, depth of injection and usually leads to pain and erythema [7]. Stem cell therapy has not been approved and studies are being carried out to concern its safety, efficacy and accessibility [8]. HF transplantation is costly and there is a shortage of donor hair follicles [9]. Therefore, there is a high demand to explore new approaches for alopecia.

HF, which contains both epithelial and mesenchymal compartment, is a complicated organ which undergoes cycles of growth (anagen), regression (catagen), quiescence (telogen) and regeneration [10]. Epithelial–mesenchymal interactions play a critical role during HF development. Dermal papilla cells (DPCs), one population of mesenchymal cells in HF, is necessary for the morphogenesis and growth of the HFs. It is also thought to be a reservoir of multipotent stem cells [11]. Dermal papilla (DP) size is thought to be well-correlated with hair cycle [12].

During the anagen phase, the cell number of DP increases and DPC acts as a signaling center to guide the surrounding epithelial cells to proliferate and migrate [13]. DPCs release various cytokines and growth factors, which are involved in the regulation of HF cycle and hair growth. It is suggested that DPCs exert their regulatory function of HF growth mainly through paracrine and autocrine [14]. Several signaling proteins, such as Wnt/β-catenin and Akt are up-regulated in DPCs after minoxidil treatment and led to the proliferation of DPCs [13].

Extracellular vesicles (EVs), composed of exosomes and microvesicles, are membranous nanovesicles (30–1000 nm in diameter) released by most cell types into the extracellular space. They can deliver internal prolific proteins, mRNAs and microRNAs into target cells and are well tolerated by the body [15]. Stem cell-derived EVs are believed to inherit the growth-promoting properties of stem cells. As previous reports, mesenchymal stem cell (MSC)-derived EVs could promote wound healing by inducing proliferation and migration of fibroblast, as well as angiogenesis in vitro [16]. According to Rajendran et al., MSC-derived EVs (MSC-EVs) have a potential to activate DP cells, prolonged survival, induce growth factor activation in vitro, and promotes hair growth in vivo [17]. It is reported that oxidized-sodium-alginate (OSA)-encapsulated EVs could increase the level of Wnt3a and β-catenin, which might be used to treat alopecia [18]. In addition, it has been shown that exosomes from bone marrow cells (BMC) prevent alopecia areata (AA) progression and sufficed for partial hair regrowth [19].These studies show that EVs may play an important role in hair growth cycle by regulating certain signaling pathway. Compared with stem cells, EVs are more stable and reservable, have no risk of aneuploidy, a lower possibility of immune rejection following in vivo allogeneic administration, and may provide alternative therapy for various diseases [20]. However, stem cells release relatively low quantities of EVs and purification of EVs is cumbersome, which results in a relatively low yield [21]. Also, the instability and low long-term retention of EVs have hindered the development of EV-based treatments.

Revealed by a previous literature, exosome-mimetic nanovesicles (NV) prepared by extruding cells can deliver therapeutic effectors [22]. The NV prepared by extruding stem cells produce 250-fold yield compared with that of exosomes [23]. Thus, NV can be an alternative strategy for clinical-scale production. Furthermore, ReNcell VM (ReN) cells, a neural progenitor cell line derived from the ventral mesencephalon region of the human fetal brain, remain stable proliferation and differentiation capacity for more than 45 passages [24]. Considering ReNcells partially maintain the characteristics of stem cells [25], we hypothesize that ReNcell-derived NV (ReN-NV) may hold potential for promoting hair growth.

In this study, ReN-NV were prepared by extruding ReN cells through serial porous membranes. On DPCs the effects of ReN-NV were observed in vitro. Using a mouse model of depilation-induced hair regeneration, ReN-NV were subcutaneously administered and the structural changes in HFs were analyzed. Furthermore, the possible underlying mechanism were investigated by Western blotting, immunofluorescence staining, and RNA sequencing. This study provides the potential for developing ReN-NV as a novel strategy for promoting hair growth.

## Results

### Preparation and characterization of ReN-NV

**Fig. 1:**
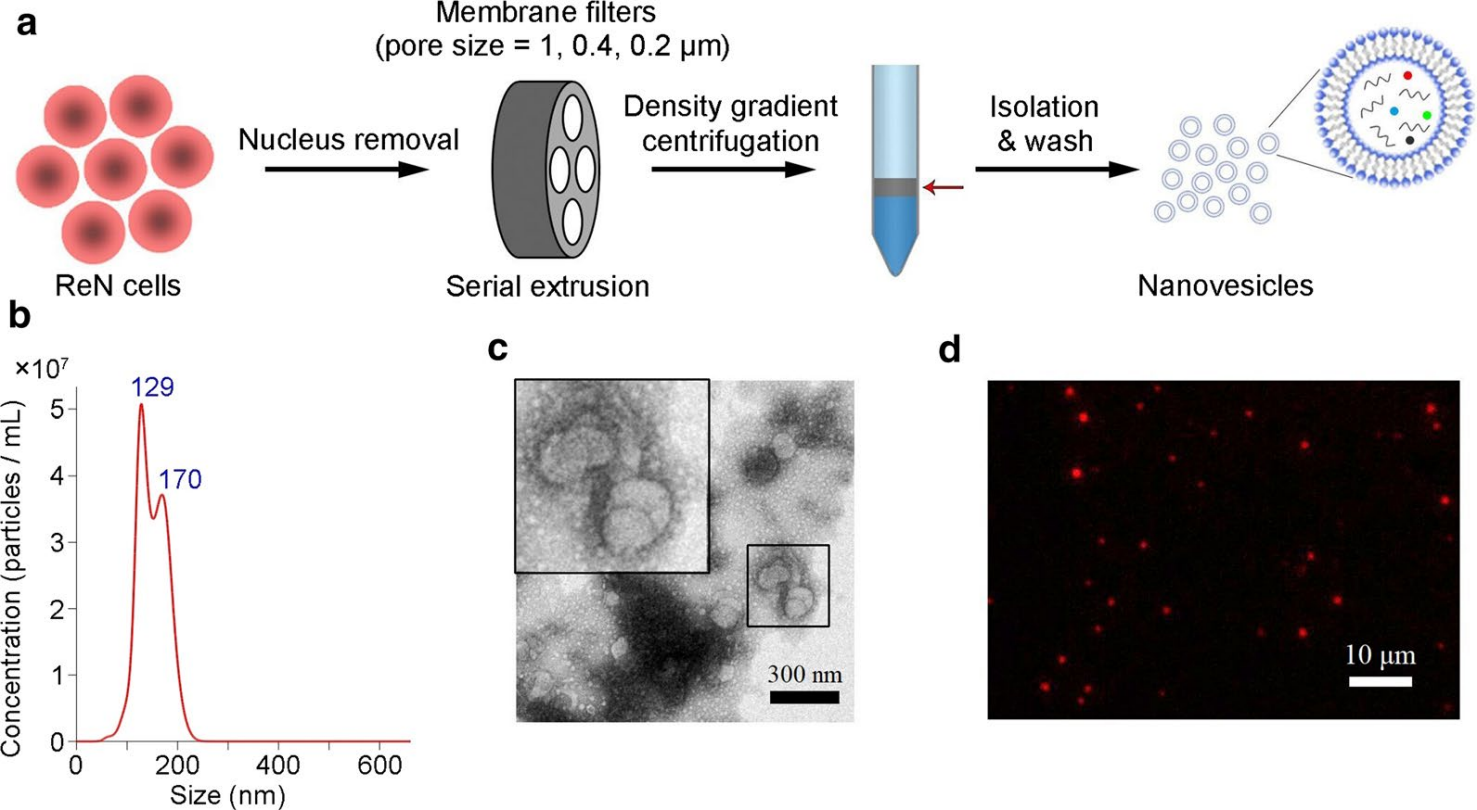
ReN cell-derived nanovesicles (ReN-NV) preparation and identification. **a** Schematic illustration of ReN-NV preparation. **b** Size distribution of ReN-NV detected by NTA measurement. **c** Transmission electron micrograph of the ReN-NV. Scale bar, 300 nm. **d** Fluorescent image of tdTomato-labeled ReN-NV (red dots). Scale bar, 10 μm

Human neural progenitor ReNcells were cultured and cell spheres formed (Additional file 1: Fig. S1). The cells were harvested and homogenated. After nucleus removal, the production was serially extruded through membrane filters with pore size of 1, 0.4 and 0.2 μm to obtain ReN-NV (Fig. 1a). NTA showed that the size of ReN-NV was < 200 nm mainly with peaks at 129 and 170 nm (Fig. 1b). TEM revealed spherical shapes in ReN-NV (Fig. 1c). In addition, to evaluate cellular uptake of ReN-NV, we labeled the membrane of ReN cells by expressing palm-tdTomato and then prepared ReN-NV. Fluorescence microscopy showed individual tdTomato-labeled ReN-NV (Fig. 1d).

### ReN-NV treatment increases DPC proliferation by β-catenin signaling pathway

**Fig. 2:**
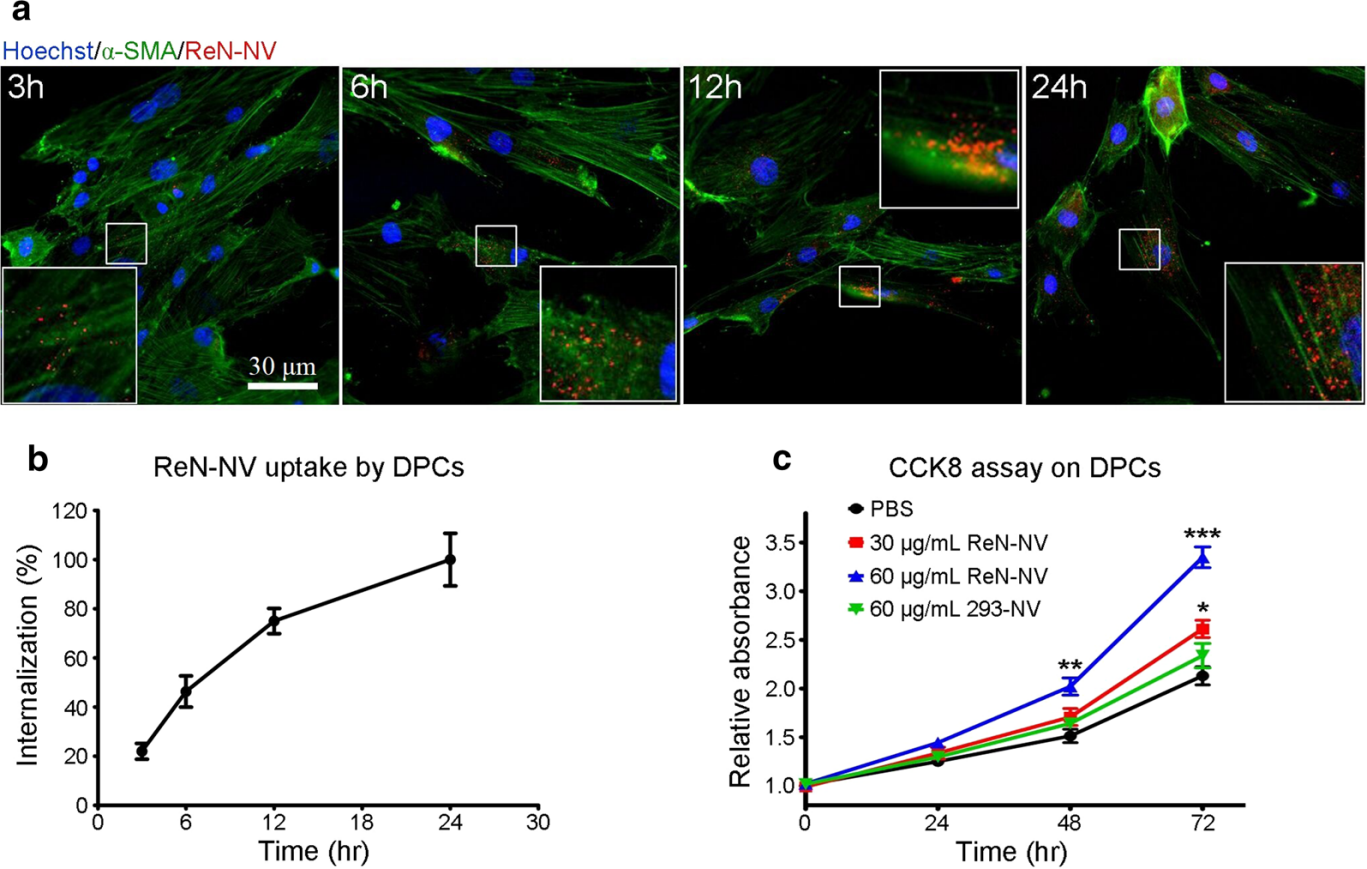
ReN-NV enter into DPCs and promote their proliferation. **a** Fluorescent images of DPCs incubated with tdTomato-labeled ReN-NV (red) for 3 h, 6 h, 12 h, and 24 h, respectively. Green represents α-SMA, Blue indicates nuclei. Scale bar, 30 μm. **b** Quantitative curve of ReN-NV uptake dynamics by determining the fluorescent intensity. **c** Cell growth curves of CCK8 assays for DPCs after treatment of PBS, ReN-NV, or 293-NV. Data are expressed as mean ± SEM. n ≥ 4; *P < 0.05, **P < 0.01, ***P < 0.001 versus the PBS groups by One-way ANOVA

Dermal papillae were isolated by improved two-step enzyme method (Additional file 2: Fig. S2). To investigate the biological effects of ReN-NV, human DPCs with passages of 3–5 were cultured and incubated with ReN-NV. After 3, 6, 12 or 24 h of incubation, DPCs were washed, fixed and immunostained with α-SMA (a marker for DPCs). Fluorescence images revealed the uptake of ReN-NV by DPCs in a time-dependent manner (Fig. 2a, b). As a control, HEK293 cell-derived NV (293-NV) were prepared and incubated with DPCs following an identical procedure. The uptake of 293-NV by DPCs was confirmed by fluorescence images (Additional file 3: Fig. S3a, b). Furthermore, the proliferation of DPCs after NV treatment was evaluated by CCK-8 assay. The proliferation of DPCs incubated with ReN-NV at the concentration of 60 μg/mL was greater than other groups (*P *< 0.001 versus PBS group at 72 h). The treatment of 30 μg/mL ReN-NV also produced significant higher cell proliferation than that of PBS at 72 h (*P *< 0.05) (Fig. 2c). These results indicate that ReN-NVs exert a promotional effect on DPC proliferation in a dose-dependent manner. To evaluate NV from other nervous tissue cells, we prepared NV by extruding human neuroblastoma SH-SY5Y cells or mouse hippocampal neuronal HT22 cells. Consistent with the results of 293-NV, the treatments of these two nervous tissue cell-derived NVs did not produce significant higher DPC proliferation than that of PBS (Additional file 4: Fig. S4).

**Fig. 3:**
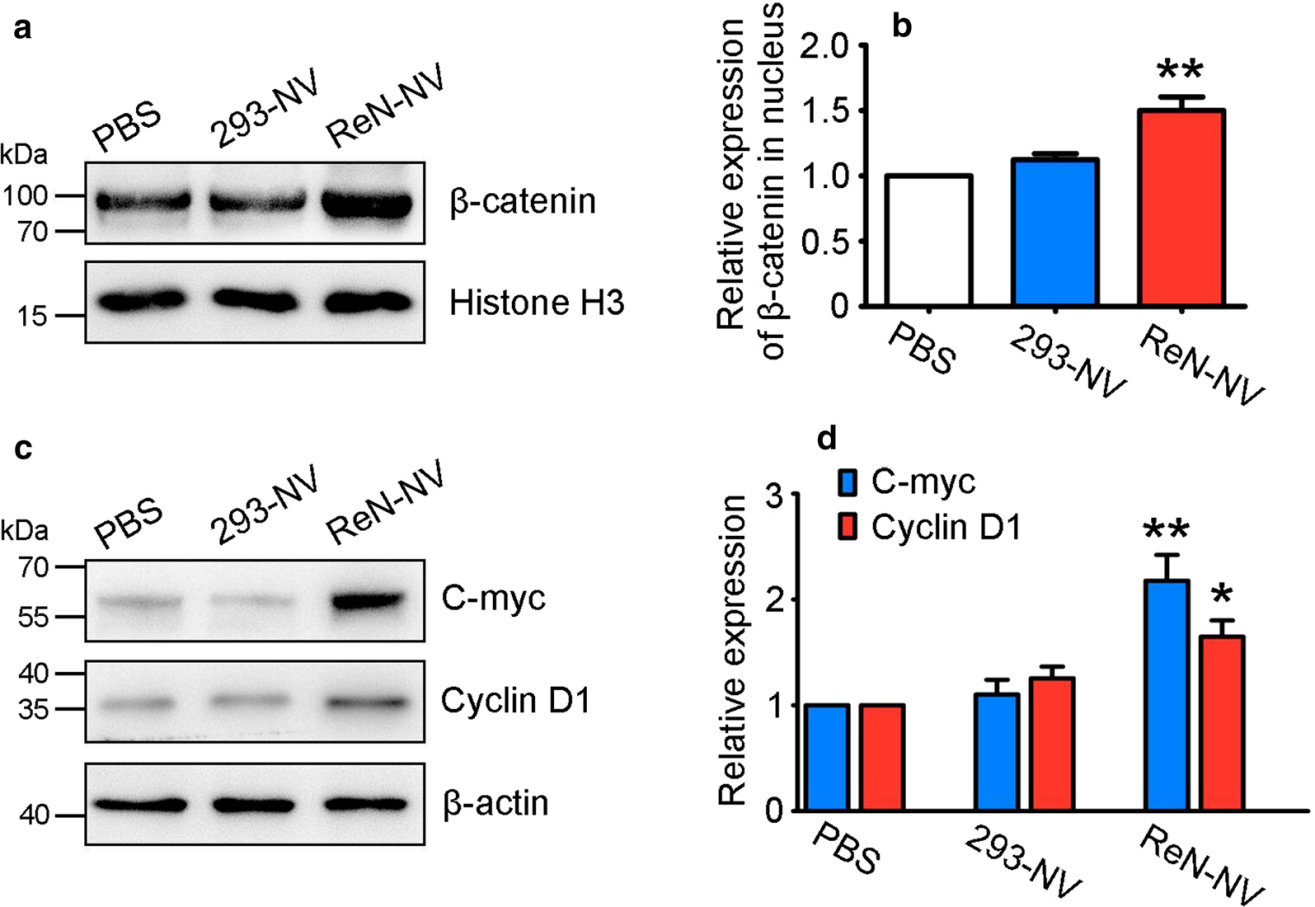
ReN-NV treatment promotes β-catenin signaling pathway in DPCs. **a** Western blotting and **b** quantitative analyses of β-catenin in nucleus were performed 48 h after treatment. **c** Western blotting and **d** quantitative analyses of C-myc and Cyclin D1 in cells were performed 48 h after treatment. Data are expressed as mean ± SEM. n ≥ 3; *P < 0.05, **P < 0.01 versus the PBS groups by One-way ANOVA

Next, to assess the effect of ReN-NVs on the hair-inductive activity of DPCs, we treated DPCs with ReN-NVs, 293-NVs and PBS, and evaluated the expression of proteins associated with anagen induction, such as β-catenin, C-myc and CyclinD1. It was showed that the nucleus expression of β-catenin in ReN-NV group was significantly higher than that in control group. While no significant change was seen between 293-NV group and control group (Fig. 3a, b). Furthermore, the expressions of C-myc and Cyclin D1, both of which were crucial downstream signaling effectors of β-catenin, were measured. Treatment with ReN-NV showed a substantial increase in C-myc and Cyclin D1 levels. However, no change was seen upon treatment with 293-NV when compared to PBS (Fig. 3c, d). Above results indicate that β-catenin signaling pathway is involved in promoting DPC proliferation activity induced by ReN-NV treatment.

### ReN-NV treatment accelerates HF growth

**Fig. 4:**
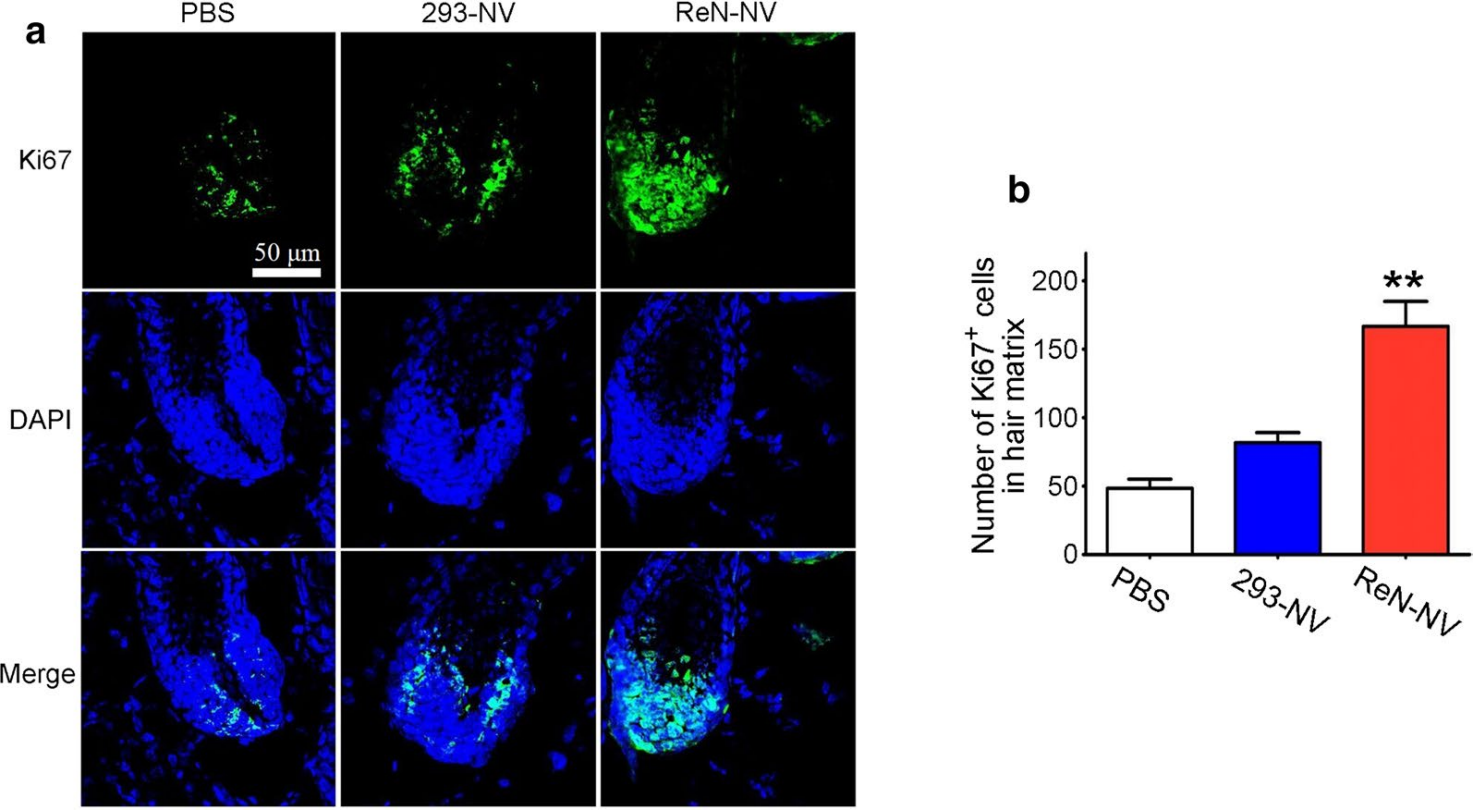
ReN-NV administration promotes cell proliferation in hair follicles (HFs). **a** Immunofluorescence images of Ki67 (green) in HFs from mice receiving depilation and 6-day subcutaneous injection (once per day). Blue indicates nuclei. Scale bar, 50 μm. **b** The number of Ki67-positive cells in hair matrix was counted. Data are expressed as mean ± SEM. n ≥ 5; **P < 0.01 versus the PBS groups by One-way ANOVA

**Fig. 5:**
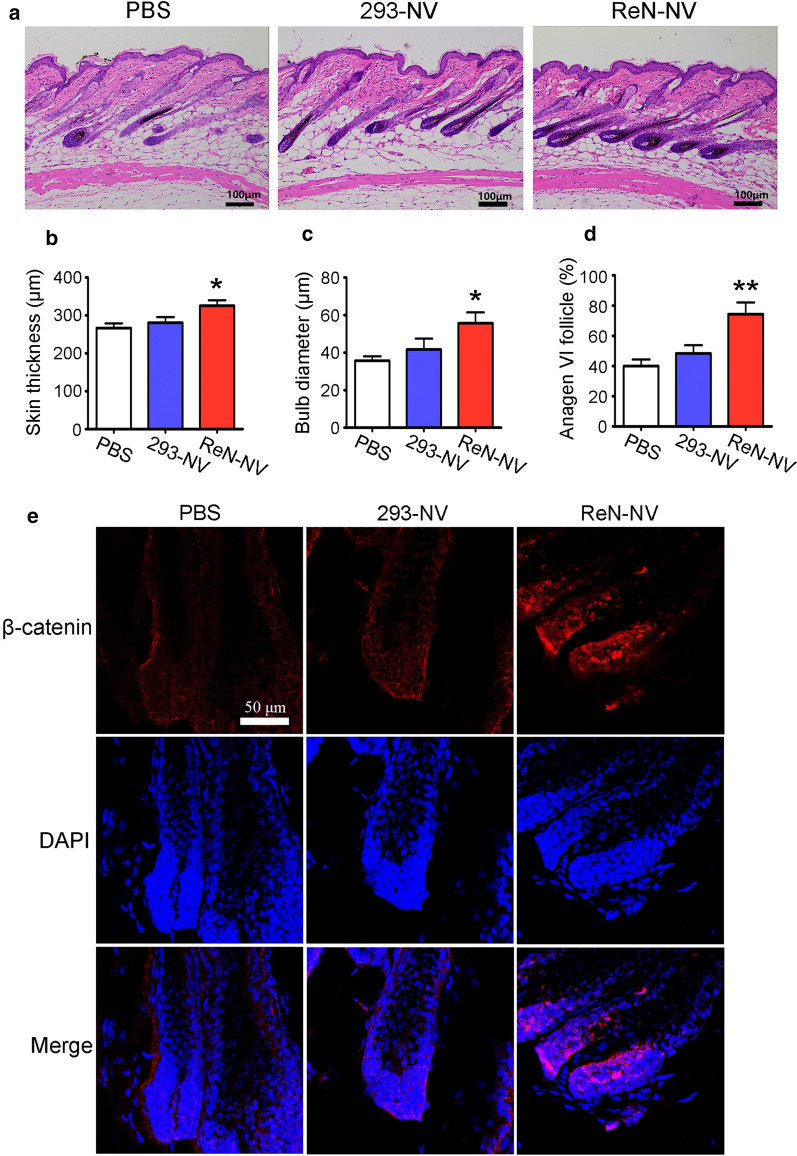
ReN-NV administration accelerates HF growth and promotes β-catenin signaling. **a** H&E staining of the skin from mice receiving depilation and 6-day subcutaneous injection (once per day). Scale bar, 100 μm. **b** The skin thickness, **c** bulb diameter and **d** anagen follicle were measured in H&E-stained sections. Data are expressed as mean ± SEM. n ≥ 5; *P < 0.05, **P < 0.01 versus the PBS groups by One-way ANOVA. **e** Immunofluorescence images of β-catenin (red) in HFs from mice receiving depilation and 6-day subcutaneous injection (once per day). Blue indicates nuclei. Scale bar, 50 μm

To study the role for ReN-NV in promoting hair growth in vivo, ReN-NV, 293-NV or PBS were injected subcutaneously in a mouse model of depilation-induced hair regeneration. Immunofluorescence staining for Ki67, a proliferation marker, was performed 6th day after the daily injection. For quantitative analyses, the number of Ki67-positive cells was counted in hair matrix keratinocytes in the hair bulb. Among the three groups, ReN-NV treatment group exhibited the highest number of Ki67-positive cells (Fig. 4a, b). In addition, The morphology of ReN-NV-treated HFs was characteristic of anagen phase, with thicker skin, larger hair bulbs and more bulbs in the subcutis (Fig. 5a). A histological analysis of H&E-stained tissue sections suggested that the skin treated with ReN-NVs was the thickest (325.4 ± 14.6 μm), compared to 293-NV (280.5 ± 15.2 μm) and PBS groups (266.6 ± 12.4 μm) (*P *< 0.05) (Fig. 5b). The hair bulb diameter of ReN-NV group (55.67 ± 5.8) was larger than 293-NV (41.67 ± 5.8) and PBS group (35.67 ± 2.3) (*P *< 0.05) (Fig. 5c). HFs in ReN-NV-treated sites were mostly in IV-VI of anagen. It was also demonstrated that 74.3% HFs treated with ReN-NV were at anagen VI stage, while 40% or 48.3% HFs in PBS or 293 group, respectively (Fig. 5d). Furthermore, the activation of β-catenin signaling pathway is essential for telogen/anagen conversion. Entry into anagen phase was more induced in mice treated with ReN-NVs, as evidenced by positive immunofluorescence staining for β-catenin (Fig. 5e). The level of β-catenin was higher in ReN-NV group compared with that in both 293-NV and PBS group. Together, these results indicate that ReN-NVs have anagen-promoting activity of the HF cycle via the regulation of β-catenin signaling pathway.

### ReN-NV significantly increases miR-100 level in DPCs

**Fig. 6:**
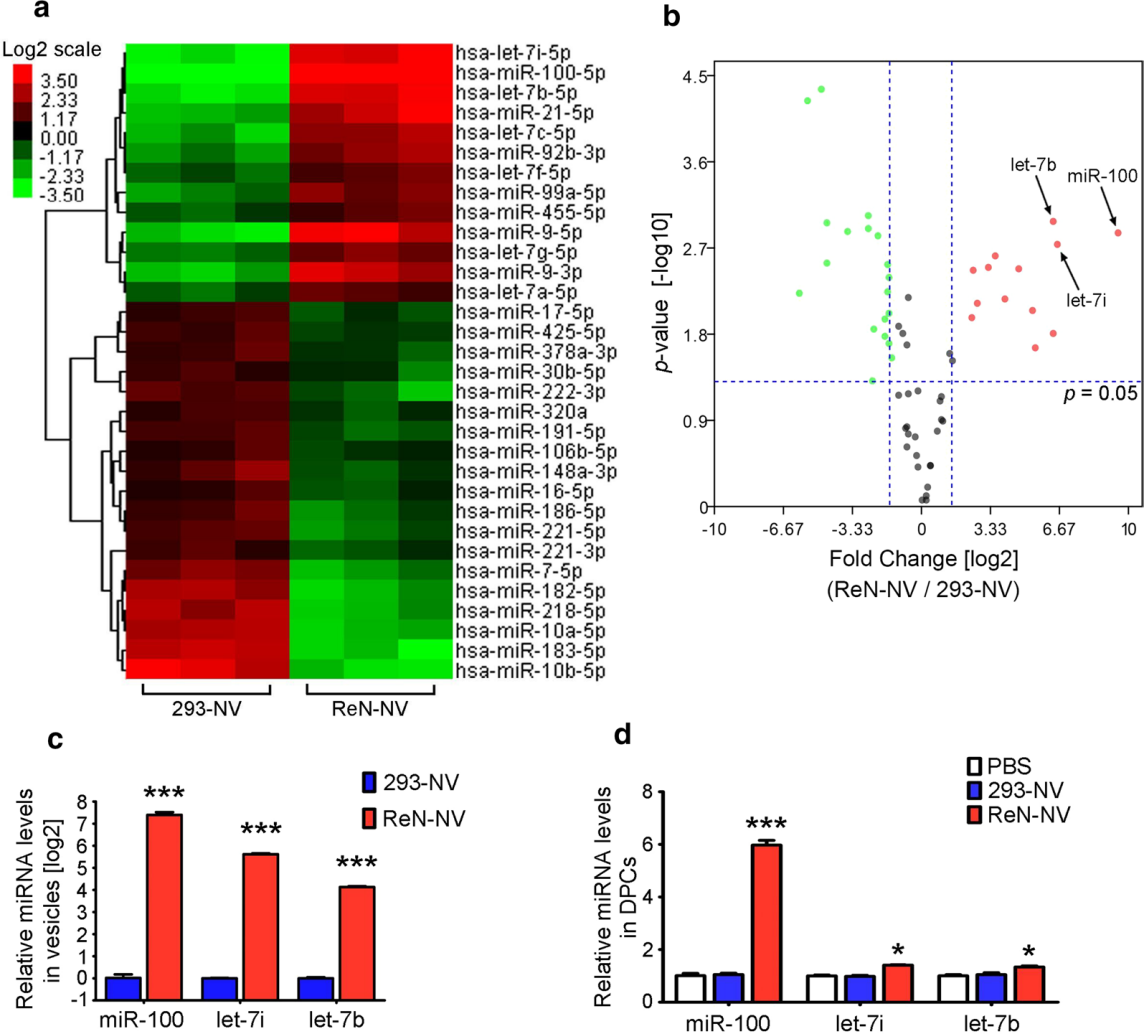
miR-100 enriched in ReN-NV. **a** Clustering and Heatmap analysis of 32 differentially packaged miRNAs in 293-NV and ReN-NV (TPM_average_ > 1000, fold change > 1.5 and *P *< 0.05). **b** Volcano plot shows the relation between the *P*-values and the fold changes. **c** RT-PCR analyses of top 3 differently packaged miRNAs in 293-NV and ReN-NV. cel-miR-39 as the external reference. **d** RT-PCR analyses of the miRNAs in DPCs were performed 24 h after the treatment of PBS, 293-NV or ReN-NV. U6 as the internal reference. Data are expressed as mean ± SEM. n ≥ 3; *P < 0.05, ***P < 0.001 versus the 293-NVgroups (**c**) or PBS groups (**d**) by One-way ANOVA

miRNAs are primary bio-regulatory molecules packaged in MSC-EVs. Given the similar size to MSC-EVs, we hypothesize that miRNAs play an important role in ReN-NV-induced HF growth. The miRNA profiles in ReN-NV and 293-NV were detected by RNA sequencing. Using TPM average > 1000, fold change > 1.5 and *P *< 0.05 as the threshold cutoff, 32 miRNAs were revealed to be significantly up- or down-regulated in ReN-NV compared with 293-NV (Fig. 6a). Subsequently, we selected three miRNAs, miR-100, let-7i and let-7b for further analysis, the expression of which showed most significant differences between ReN-NV and 293-NV (Fig. 6b). The up-regulation of these miRNAs was confirmed by RT-PCR (fold change = 168.6, 49.1 and 17.5, respectively) (Fig. 6c). Furthermore, we evaluated the amount of the miRNAs in DPCs after incubation with PBS, 293-NV or ReN-NV. Compared with PBS treatment, ReN-NV treatment resulted in remarkable increases in all the three miRNA levels in DPCs, while 293-NV produced no significant change (Fig. 6d). Interestingly, the highest fold change is observed for miR-100 with a 6.0-fold change compared with 1.4 for let-7i and 1.3 for let-7b. These results show that miR-100 is significantly abundant in ReN-NV and delivered to DPCs following the uptake of ReN-NV. It is hypothesized that miR-100 might be involved in ReN-NV-induced HF growth.

### ReN-NV-derived miR-100 promotes β-catenin signaling pathway

**Fig. 7:**
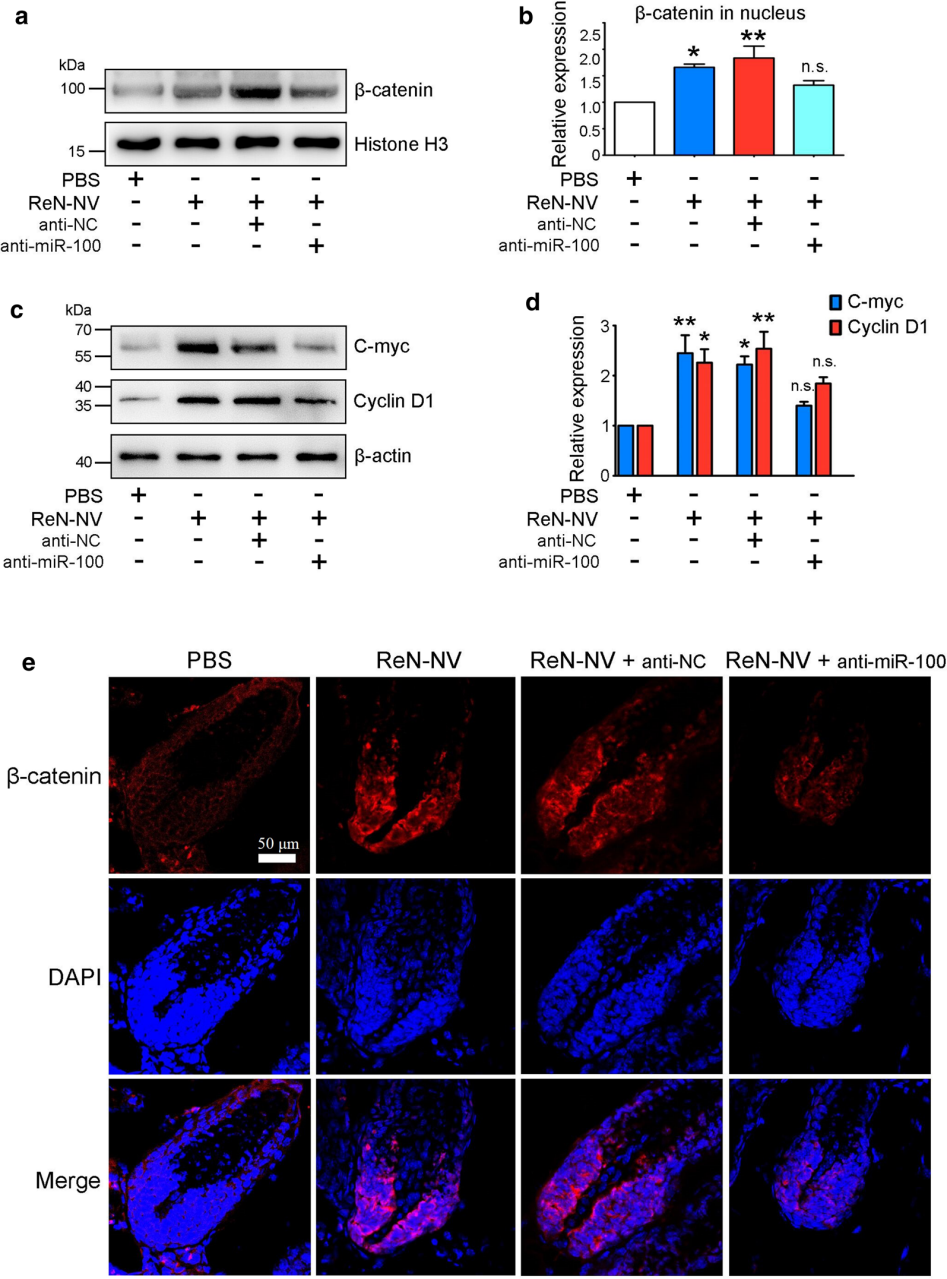
ReN-NV promote β-catenin signaling pathway through miR-100. **a** Western blotting and **b** quantitative analyses of β-catenin in nucleus were performed 48 h after treatment. **c** Western blotting and **d** quantitative analyses of C-myc and Cyclin D1 in cells were performed 48 h after treatment. Data are expressed as mean ± SEM. n ≥ 3; *P < 0.05, **P < 0.01, *n.s.*, no significance versus the PBS groups by One-way ANOVA. **e** Immunofluorescence images of β-catenin (red) in HFs from mice receiving depilation and 6-day subcutaneous injection (once per day). Blue indicates nuclei. Scale bar, 50 μm

According to previous reports, miR-100 repress multiple β-catenin negative regulators and increase β-catenin signaling. To elucidate the biological function of miR-100 delivered by ReN-NV, synthetic anti-miR-100 was transfected 12 h before the co-incubation of DPCs and ReN-NV, and then Western blottings were performed to access β-catenin expression in nucleus. Intervention with anti-miR-100 suppressed the ReN-NV-induced up-regulation of β-catenin level (Fig. 7a, b). Consistent with the trend of β-catenin, the expressions of C-myc and CyclinD1 were increased by ReN-NV and this effect was impaired when anti-miR-100 was applied (Fig. 7c, d). Next, to confirm the role of miR-100 in β-catenin up-regulation induced by ReN-NV in vivo, cholesterol-conjugated anti-miR-100 was loaded into ReN-NV and the modified ReN-NV were injected subcutaneously in a mouse model of depilation-induced hair regeneration. After daily injection for 6 consecutive days, immunofluorescence staining for β-catenin was carried out. Increased fluorescent signals in the hair bulbs were observed after ReN-NV injection compared with that under PBS treatment. However, the increase was suppressed when anti-miR-100 was loaded (Fig. 7e). Taken together, these results indicate that ReN-NV-derived miR-100 enhances the nucleus expression of β-catenin, thereby increasing C-myc and Cyclin D1 levels, resulting in the acceleration of HF growth.

## Discussion

In this study, we aimed to obtain evidences for direct roles of ReN-NV in hair growth. DPCs, derived from mesenchymal components, are capable of releasing growth factors that direct epithelial cells to proliferate. Thus, DPCs are considered to be a key signaling center responsible for the induction and maintenance of follicular development, regulation of the hair cycle, as well as the regeneration of HFs [26]. First of all, we have successfully fabricated ReN-NV from ReN cells using a serial extrusion through membrane filters with diminishing pore sizes (1, 0.4, 0.2 μm), and the production was identified to be in accordance with the previous reports [22]. Then, the biological function of ReN-NV on DPCs was investigated and the underlying mechanisms were explored. Using fluorescently labeled ReN-NV, we confirmed that their uptake by DPCs in a time-dependent manner when incubate them with cells. In addition, we found that ReN-NV promoted the proliferation of DPCs as determined by CCK-8 analysis. As well known, cell cycle control is important for cell proliferation. β-catenin, a regulator of many gene such as CyclinD1 and C-myc, plays an important role in cell cycle progression. Also, β-catenin accumulates in the cytoplasm and then translocates into the nucleus, leading to increased expressions of downstream molecules. Here, ReN-NV were revealed to have the capacity to promote DPC proliferation. The mechanism may be due to the up-regulation of β-catenin signaling pathway.

To study the effect of ReN-NV in vivo, C57BL/6 mouse is an ideal model animal to observe hair cycle, since the dorsal hair in these mice has a time-synchronized growth cycle. During telogen phase, the depilated skin of C57BL/6 mice is pink, while it darkens at initiation of anagen [27]. The HFs in the anagen phase exhibited typical features, such as a larger hair bulb, thicker skin, more bulbs in the subcutis, as well as larger amounts of melanin [9]. Herein, we investigated the effect of ReN-NV on hair re-growth in a mouse model of depilation-induced hair regeneration. As shown in our study, ReN-NV was injected subcutaneously once a day. After injection for 6 consecutive days, sections from dorsal skin of mice revealed that ReN-NV group had the greatest number of Ki67-positive cells, which expressed mostly in hair bulb, compared to 293-NV and control groups. The expression level of Ki67 in hair matrix area is an important marker to assess the proliferation of hair follicle cells [28]. These data indicated that ReN-NV might enhance hair follicle cells proliferation and promote telogen-to-anagen transition of hair follicles. Furthermore, a histological analysis of H&E-stained tissue sections demonstrated that the thickest skin, largest bulb diameter and highest percentage of anagen VI HFs could be found in HFs treated with ReN-NVs, exhibiting that ReN-NVs significantly induced earlier anagen entry and accelerated hair follicle growth compared with control group.

miRNA profiling analysis revealed that miR-100 was much more abundant in ReN-NV than 293-NV. It was striking that miR-100 in DPCs was up-regulated as high as sixfold after incubation with ReN-NV. In previous studies, miR-100 plays a critical role in tissue regeneration. Aguirre et al. identified miR-99/100 and let-7a/c were critical regulators of cardiomyocyte regeneration, which improve heart function recovery after injury. It is also demonstrated that miR-100 promotes MSC osteogenesis [29]. Recently, miR-100 activate Wnt signaling pathway by repressing multiple Wnt negative regulators, along with increased nuclear β-catenin expression [30]. It is well known that Wnt/β-catenin signaling pathway is crucial for the regeneration of hair cycle, abnormity of which may cause hair loss of different types. In addition, the maintenance of the anagen phase is dependent on β-catenin [28]. However, there is few study focus on the interaction between miR-100 and hair follicle cells. Based on the above mentioned studies, we speculated that miR-100 may play an important role in ReN-NV promoting proliferation of DPCs through Wnt/β-catenin signaling pathway. To further confirm the role of miR-100, anti-miR-100 was applied together with ReN-NV in vitro and in vivo. miR-100 knock down significantly suppressed ReN-NV-induced increases of β-catenin, Cyclin D1 and C-myc. Taken together, miR-100 in ReN-NV promoted the proliferation of dermal papilla cells by up-regulation of Cyclin D1 and C-myc via the activation of β-catenin pathway.

Previous studies focus on the facilitation of hair regrowth using EVs or exosomes. Hu et al. prepared an oxidized sodium alginate hydrogel encapsulating dermal papilla cell-derived extracellular vesicles (DP-EVs) [18]. The release system facilitated hair regrowth in vivo and in vitro resulting from upregulation of hair growth-promoting signaling molecules such as β-catenin and Wnt3a, and downregulation of inhibitory molecule BMP2. Their work could be a great improvement for the long term therapeutic use of EVs. However, DPC is not a reliable and expandable cell source for large-scale production of EVs. Furthermore, Cheng et al. revealed that miR-218-5p-overexpressed exosomes accelerated the onset of anagen, and DPC spheroid culture provided a benificial avenue for cell therapy [31]. The authors also mentioned that the lack of hormone-related or disease-related hair loss model needed to be filled up in the future. Different from exosomes, which are released by cells, NVs are prepared by extruding cells. According to a proteomic study, NV proteins largely mirror their parental cells, while exosomal proteins mainly from their endosomal origin [32]. This advantage of NV bypasses the obstacle of endosomal sorting of proteins or RNA into exosomes. Here, our data revealed the great promise of NV to promote hair growth. In addition, some current reports revealed that NVs are a potentially promising alternative to exosomes for clinical applicability, given their higher yield without incumbent production [33–35].

## Conclusions

In summary, NV were prepared from stable ReN cell line by serial extrusion and found to promote DPC proliferation and HF growth. The underlying mechanism was indicated to be the activation of Wnt/β-catenin signaling pathway mediated by ReN-NV-derived miR-100. This study provides the potential for developing ReN-NV as a novel strategy for promoting hair growth. In the future, it could be beneficial for large-scale production of ReN-NV for further studies and non-scaring alopecia therapies. In addition, other drug delivery system, such as microneedle patch, could be used to improve treatment compliance of ReN-NV.

## Materials and methods

### Isolation and identification of ReN-NV

ReN cells were purchased from Millipore (USA). The cells with passages of 5–10 were cultured in DMEM/F12 (Life Technologies, USA) supplemented with 2% B27 (Life Technologies), 20 ng/mL EGF (Abm, Canada), 10 ng/mL bFGF (Abm) and 2 μg/mL heparin and incubated with 5% CO_2_ at 37 °C. ReN cell spheres were dissociated with Accutase (Life Technologies). The cells were centrifuged at 3000×*g* for 10 min to remove the nucleus after homogenate by ultrasonication. The supernatants were extruded serially through 1 μm, 400 nm and 200 nm polycarbonate porous membranes (Whatman, UK) using a mini extruder (Avanti Polar Lipids, USA). Subsequently, the extruded ReN-NV were purified in a layer between 10% and 50% (v/v) iodoxanol by ultracentrifugation (L-80XP, Bechman Coulter, USA) at 100,000×*g* for 2 h. The ReN-NV pellet was resuspended in PBS after a wash step by another ultracentrifugation with PBS.

ReN-NV were observed by a Tecnai G2 transmission electron microscope (FEI, USA). The total protein concentration was determined by BCA protein assay (Pierce, USA). The size distribution and number were analyzed by NTA using a ZetaView system (Particle Metrix, Germany). As a control, HEK293 cell-derived NV (293-NV) were prepared and identified following an identical procedure.

### Isolation and culture of human DPCs

The study using clinical samples was approved by the Review Board of Nanjing Medical University (2018143). Healthy human scalp specimens were obtained with informed consent from subjects undergoing cosmetic surgery. Improved two-step enzyme method was used as described previously to isolate the dermal papillae [36]. In brief, the skin was sterilized and digested in 0.5% (w/v) dispase (Sigma, USA) for 12–16 h at 4 °C and in 0.2% (w/v) collagenase D (Sigma, USA) for 6 h at 37 °C sequentially. The digested tissue was then centrifuged at 550–850*g* for 5 min. After centrifuged for 3 times, the human dermal papillae were separated from other types of cells. The harvested DPCs were cultured in DMEM (Life Technologies) containing 10% heat-inactivated fetal bovine serum (Life Technologies) and 1% Pen Strep, then incubated at 37 °C in an atmosphere of 95% air and 5% CO_2_.

### The cellular uptake of ReN-NV

To label ReN-NV with tdTomato, ReNcells were stably transduced with packaged lentivirus vectors to express tdTomato fused to palmitoylation signal (palm-tdTomato) which labels cell membrane [37]. The tdTomato-labeled ReN-NV were prepared by extruding the cells, and observed using a Ti-E fluorescence microscope with a 100× objective (Nikon, Japan). DPCs were incubated with 30 μg/mL tdTomato-labeled ReN-NV at 37 °C. At given time points, the cells were washed by PBS and immunostained with anti-α-SMA (Santa Cruz, USA) and Alexa 488-conjugated secondary antibodies (Life Technologies). After staining by DAPI (Sigma, USA), the cells were imaged by fluorescence microscope with a 20× objective (Nikon). Images were processed and analyzed by ImageJ software (NIH, USA). All settings of imaging and processing were kept constant, and the relative fluorescence intensities were calculated.

### Cell proliferation assay

Cell proliferation rates were measured by using a Cell Counting Kit-8 (CCK-8) (Dojindo, Japan). DPCs (5 × 10^4^ cells/well) were seeded in a 96-well plate and incubated with PBS, ReN-NV (30 or 60 μg/mL) or 293-NV (60 μg/mL). Cell proliferation of each group was calculated at the time point of 24, 48 and 72 h, respectively. CCK-8 reagent (10 μL) was added to each well and incubated for 2 h. Then, O.D. 450 nm value in each well was verified by a microplate reader. The experiment was repeated three times.

### Antisense miRNAs

The antisense nucleotide of miR-100 (anti-miR-100) and negative control (anti-NC) were synthesized and modified with 2′Ome by GenePharma (China). The sequences were as follows: 5′-CACAAGUUCGGAUCUACGGGUU-3′ for anti-miR-100, 5′-CAGUACUUUUGUGUAGUACAA-3′ for anti-NC. DPCs were transfected with 100 nM antisense miRNAs in 6-well plates with lipo2000 (Life Technologies) according to manufacturer’s instructions, followed by ReN-NV treatment 12 h later. To apply antisense miRNAs in vivo, anti-miR-100 and anti-NC were conjugated with cholesterol on the 3′ terminus (GenePharma), and then loaded into ReN-NV following our previous study [38]. Briefly, 100 nM cholesterol-conjugated anti-miR-100 or anti-NC were incubated with 100 μg ReN-NV in 200 μL of PBS at 37 °C for 1 h. The antisense miRNAs inserted into ReN-NV membrane through a hydrophobic interaction. After washing with PBS at 140,000*g* for 90 min, the modified ReN-NV were resuspended and stored at − 80 °C prior to use.

### Western blotting analysis

Cells or tissues were lysed or homogenized in RIPA buffer with protease inhibitors (Pierce, USA). After centrifugation at 12,000 rpm for 30 min at 4 °C, the supernatants were loaded in 10% SDS-PAGE gel for electrophoresis (Bio-Rad, USA).Then the samples were transferred onto PVDF membrane (Millipore) and incubated with antibodies as follows: anti-β-catenin, anti-C-myc, and anti-Cyclin D1 were from Abcam (UK). After incubation with HRP-conjugated secondary antibodies, the signals were visualized using ECL Substrates (Bio-Rad). Histone H3 or β-actin served as the control group.

### Immunofluorescence staining

For immunofluorescence assay, the mouse skin was isolated and cryosectioned in 5-μm thickness. The sections were treated with 0.3% Triton X-100 for 30 min and 3% BSA for 2 h, and immunostained with anti-β-catenin (Abcam, UK) or anti-Ki67 (Cell Signaling Technology, USA) at room temperature (RT) for 2 h. After washing 5 times by PBST (PBS containing 0.1% Triton X-100), the samples were incubated with Alexa 488 or Alexa 594-conjugated secondary antibodies (Life Technologies) for 1 h at RT. After washing 5 times by PBST, staining by DAPI, and mounting with ProLong Diamond Antifade Mountant (Life Technologies), the tissue slides were imaged by an FV-1200 confocal microscope (Olympus, Japan). Images were processed and analyzed by ImageJ software (NIH). All settings of imaging and processing were kept constant.

### Animals

Six-week-old female C57BL/6 mice were purchased from the Animal Core Facility of Nanjing Medical University (Nanjing, China). All animal experiments were carried out in compliance with institutional guidelines and were approved by the Animal Care and Use Committee of Nanjing Medical University (IACUC-1910003). Mice were randomly divided into the Control (PBS, n = 5), 293-NV (n = 5) and ReN-NV (n = 5) groups. The 4 injection spots (25 μL per site) were evenly distributed on the dosal skin, spaced 1 cm apart. A total of 100 micro-litre of PBS, 293-NV or ReN-NV were subcutaneously injected once daily into the dorsal skin of C57BL/6 mice from depilation day (p.d.) 0. The mice sacrificed at p.d. 6. The dorsal skin samples were harvested for further analysis as previously described [39].

### Quantitative histomorphometry

To investigate the progression of HFs in the hair cycle, we performed H&E staining of skin biopsy sections. The dorsal skin tissue fixed in 4% paraformaldehyde was embedded in paraffin and then cut into sections at a thickness of 5 μm, followed by staining with hematoxylin and eosin. In histological analysis, measurement of skin thickness, bulb diameter and anagen VI HF percentage is a well-known method to classify the hair cycle stages [26]. The ImageJ software (NIH) was used for quantitative analyses. Skin thickness was measured as the distance from the epidermis to the subcutaneous fat under microscopic field. Measurements were carried out in three fields per mouse and the average value was expressed in micrometers. Bulb diameter was measured at the level of the largest diameter (“Auber’s line”) of the hair bulbs with clearly visible DP. Anagen VI HF percentage = number of anagen VI HF/number of total HF × 100%. At least 50 HFs per sample were analyzed.

### miRNA sequencing and analysis

For miRNA sequencing, RNA was isolated by Trizol (Life Technologies) extraction from ReN-NV and 293-NV. Qubit 2.0 and Agilent 2100 bioanalyzer were used to quantify the samples. The cDNA libraries were produced using a NEBNext Ultra small RNA Sample Library Prep Kit for Illumina according to the manufacturer’s instructions. Subsequently, the library preparations were sequenced on an Illumina Hiseq 2500 platform and paired-end reads were generated. Using Bowtie software, the clean reads were compared with Silva database, GtRNAdb database, Rfam database and Repbase database sequence alignment to filter ribosomal RNA(rRNA), transfer RNA (tRNA), small nuclear RNA (snRNA), small nucleolar RNA (snoRNA)and other ncRNA and repeats. The remaining reads were used to detect known miRNA and new miRNA predicted by comparing with known miRNAs from miRBase. The miRNA levels were calculated and normalized to transcripts per million (TPM). A heatmap analysis of miRNA expression levels was created based on the TPM values of miRNAs in ReN-NV and 293-NV (using TPM_average_ > 1000, 1.5-fold change and P < 0.05 as the threshold cutoff).

### RT-PCR for miRNA evaluation

Total RNA was extracted from ReN-NV, 293-NV and cells using Trizol reagent. For NV RNA extraction, 100 μgNV were used with 10 femto-moles cel-miR-39 as an external reference and a RNeasy MinElute Spin Column (Qiagen, Germany) was employed. To detect miRNAs, miRcute Plus miRNA First-Strand cDNA Kit, qPCR Kit and primers (TIANGEN, China) were used following the manufacturer’s instructions. The results were normalized to cel-miR-39 or U6 snRNA levels. Relative expression was calculated by the comparative 2^−ΔΔCt^ method. All experiments were performed at least three times independently.

### Statistical analysis

All data were analyzed statistically using GraphPad Prim software (USA). Results were expressed as mean ± SEM after at least three independent tests. The significances among groups were analyzed using the Student’s *t*-test or One-way ANOVA. *P* value < 0.05 was regarded as statistical significant.

## Supplementary information

**Additional file 1: Fig. S1.** Microscopic image of ReNcell spheres.

**Additional file 2: Fig. S2.** Microscopic image of DPCs.

**Additional file 3: Fig. S3.** a) Fluorescent images of DPCs incubated with tdTomato-labeled 293-NV (red) for 3 h, 6 h, 12 h, and 24 h, respectively. Green represents α-SMA, blue indicates nuclei. Scale bar, 30 μm. b) Quantitative curve of 293-NV uptake dynamics by determining the fluorescent intensity. Data are expressed as mean ± SEM. n ≥ 4.

**Additional file 4: Fig. S4.** Cell growth curves of CCK8 assays for DPCs after treatment of PBS, HT22-NV, or SH-SY5Y-NV. Data are expressed as mean ± SEM. n ≥ 4.

## Data Availability

All data generated or analyzed during this study are included in this published article.

## Abbreviations

EV: Extracellular vesicle
NV: Nanovesicle
DPC: Dermal papilla cell
HF: Hair follicle
DP: Dermal papilla cell
miR-100: MicroRNA-100
MSC: Mesenchymal stem cell
OSA: Oxidized-sodium-alginate
