# Supplementary figures and images for: Neural progenitor cell-derived nanovesicles promote hair follicle growth via miR-100
Source: J Nanobiotechnology. 2021 Jan 11;19:20. doi: 10.1186/s12951-020-00757-5 (PMC7802142; doi:10.1186/s12951-020-00757-5)

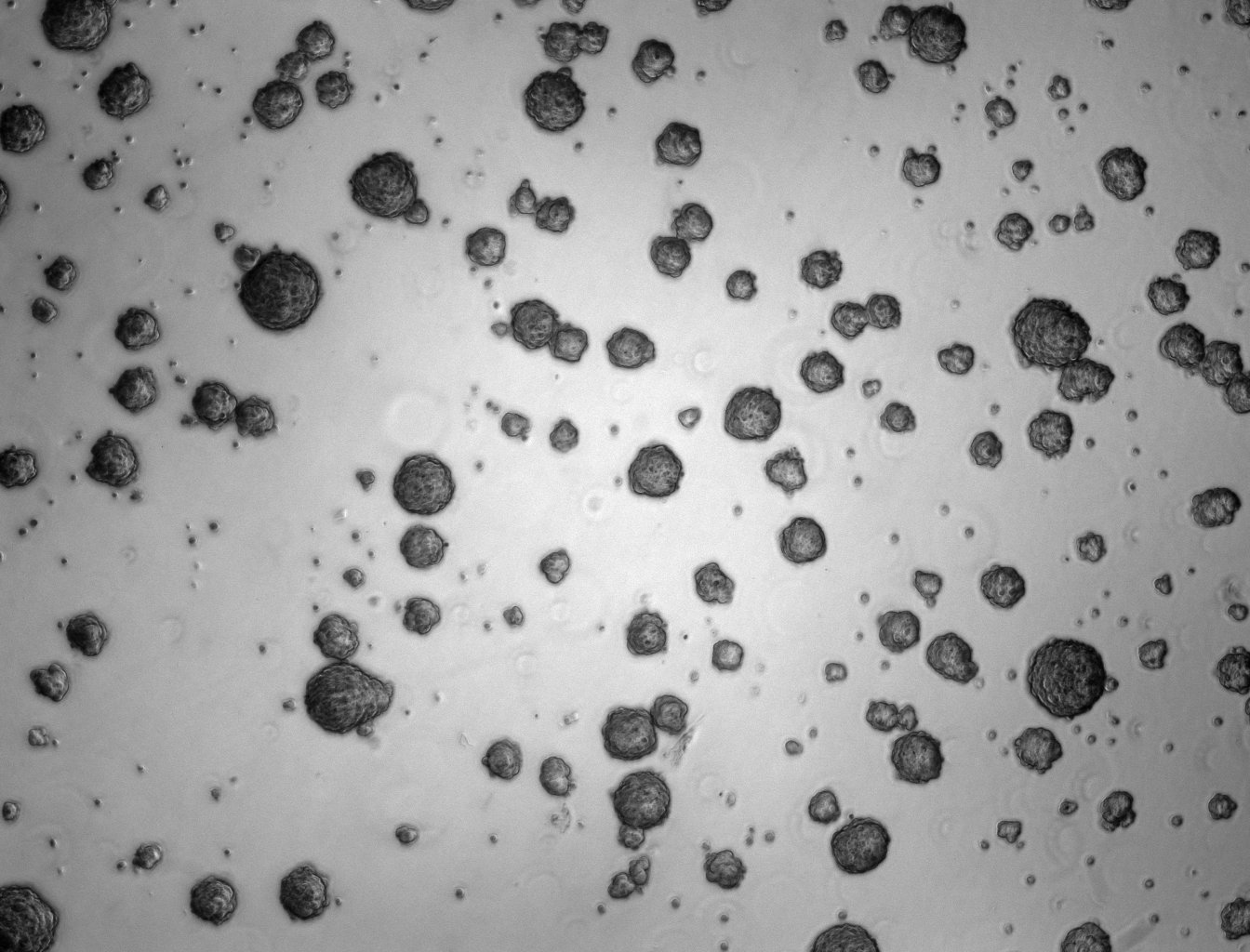

Supplement: Supplementary file 1 — Additional file 1: Fig. S1. Microscopic image of ReNcell spheres. [file 12951_2020_757_MOESM1_ESM.jpg]

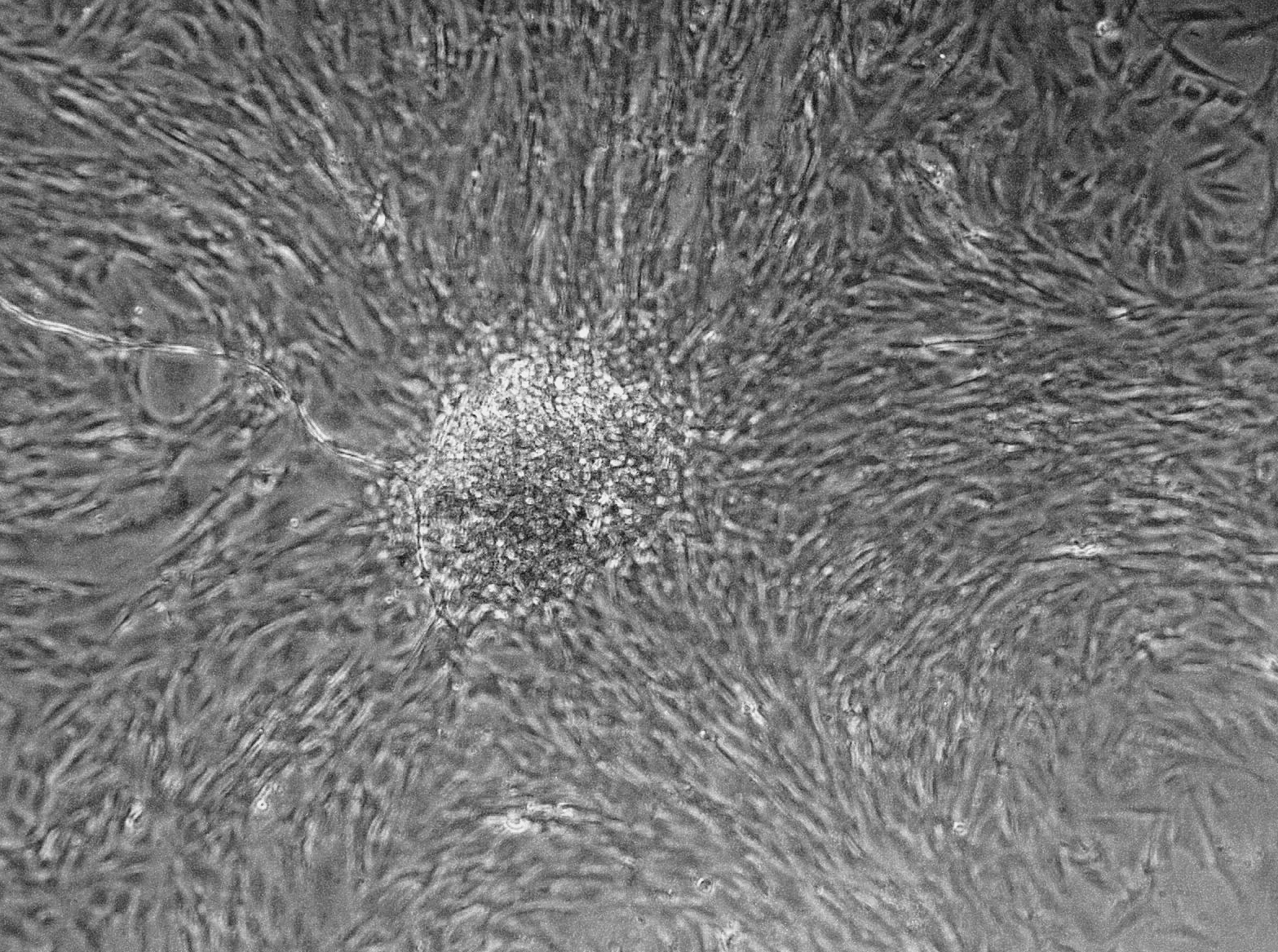

Supplement: Supplementary file 2 — Additional file 2: Fig. S2. Microscopic image of DPCs. [file 12951_2020_757_MOESM2_ESM.jpg]

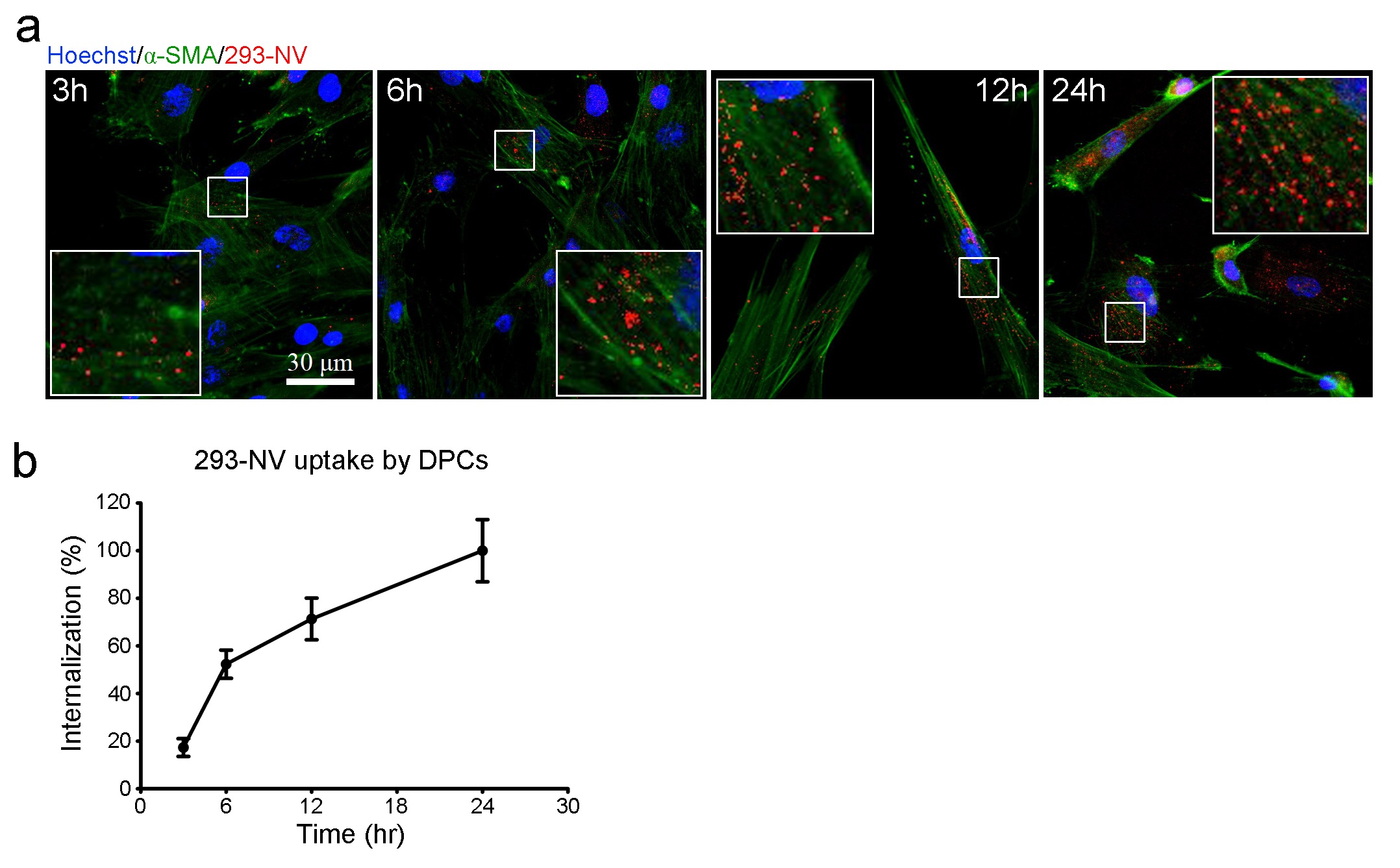

Supplement: Supplementary file 3 — Additional file 3: Fig. S3. a) Fluorescent images of DPCs incubated with tdTomato-labeled 293-NV (red) for 3 h, 6 h, 12 h, and 24 h, respectively. Green represents α-SMA, blue indicates nuclei. Scale bar, 30 μm. b) Quantitative curve of 293-NV uptake dynamics by determining the fluorescent intensity. Data are expressed as mean ± SEM. n ≥ 4. [file 12951_2020_757_MOESM3_ESM.jpg]

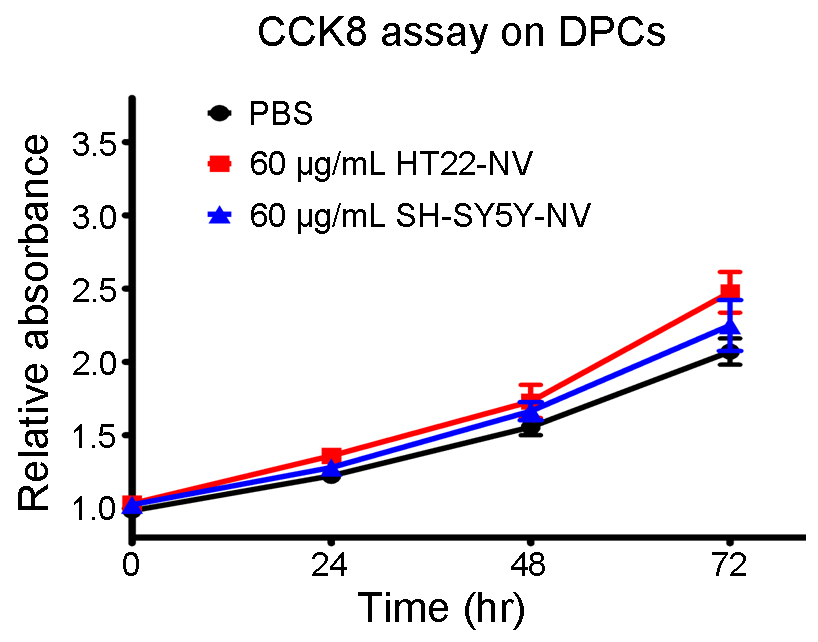

Supplement: Supplementary file 4 — Additional file 4: Fig. S4. Cell growth curves of CCK8 assays for DPCs after treatment of PBS, HT22-NV, or SH-SY5Y-NV. Data are expressed as mean ± SEM. n ≥ 4. [file 12951_2020_757_MOESM4_ESM.jpg]
